# Takotsubo Cardiomyopathy in a Squash Player

**DOI:** 10.4061/2009/351621

**Published:** 2009-07-21

**Authors:** Mathieu Berry, Jerome Roncalli, Olivier Lairez, Meyer Elbaz, Didier Carrié, Michel Galinier

**Affiliations:** Department of Cardiology, Rangueil University Hospital, 31059 Toulouse, France

## Abstract

Takotsubo cardiomyopathy is usually described following acute emotional stress. We report here the case of a 48-year-old woman admitted for acute coronary syndrome after an intensive squash match. Diagnosis of Takotsubo cardiomyopathy due to acute physical stress was suspected in presence of normal coronary arteries and transitory left ventricular dysfunction with typical apical ballooning. Cardiac magnetic resonance imaging confirmed regional wall-motion abnormalities and was helpful in excluding myocardial infarction diagnosis. During squash the body is subject to sudden and vigorous demands inducing a prolonged and severe workload on the myocardium.

## 1. Introduction

Takotsubo cardiomyopathy is a heart syndrome associated with transient contractile dysfunction. Takotsubo cardiomyopathy is usually described following acute emotional stress. We report here a typical Takotsubo syndrome after an intensive squash practice.

## 2. Case Report

**Figure 1:**
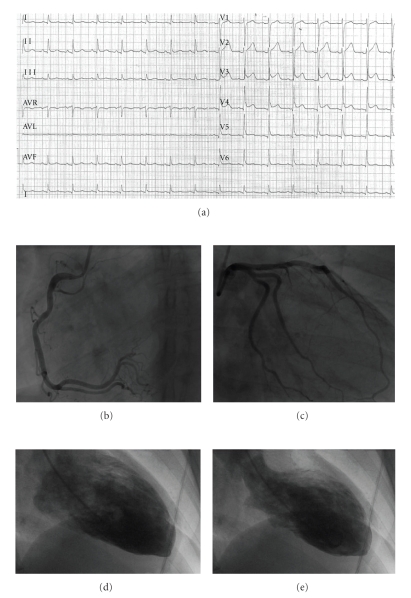
Acute coronary syndrome with angiographically normal coronary arteries and apical ballooning of the left ventricle. (a) Twelve-lead ECG demonstrating ST-segment elevation in anterior leads; (b) normal right coronary artery; (c) normal left coronary artery; (d) contrast left ventriculography in the right anterior oblique projection at end-diastole; (e) end-systole showing apical ballooning and basal hyperkinesia.

A 48-year-old woman was admitted to our emergency department for chest pain. She mentioned no past of coronary artery disease but a 40-pack year smoking history. Drug intake was levothyroxine since a thyroidectomy was performed for a Grave's disease. Her main hobby consisted of practicing squash one day a week. During a third intensive match in a one-day tournament, she reported a significant unusual breathlessness. Five minutes after the match, during the recovery time, she complained of a sudden intensive and substernal constrictive chest pain. Due to persistent symptoms (about two hours), the woman went to the hospital emergency department, where an electrocardiogram (ECG) revealed ST-segment elevation in the anterior leads (Figure 1(a)).

At admission, she was eupnoeic with a blood pressure of 130/90 mmHg, and a pulse heart rate of 90 beats/min. Clinical examination was normal.

She was immediately transferred to our catheterization laboratory in suspicion of acute coronary syndrome (ACS) with ST-segment elevation myocardial infarction. Urgent coronary angiography was performed and revealed no coronary stenosis (Figures 1(b) and 1(c)). However, right anterior oblique ventriculography showed characteristic regional wall-motion abnormalities involving a typical apical ballooning (Figures 1(d)  and 1(e)).

Troponin T level was increased (6.7 ng/mL). All other blood tests including hemoglobin, coagulation parameters, glucose, creatinine, serum electrolytes, C-reactive protein, and liver function variables were normal. 

**Figure 2:**
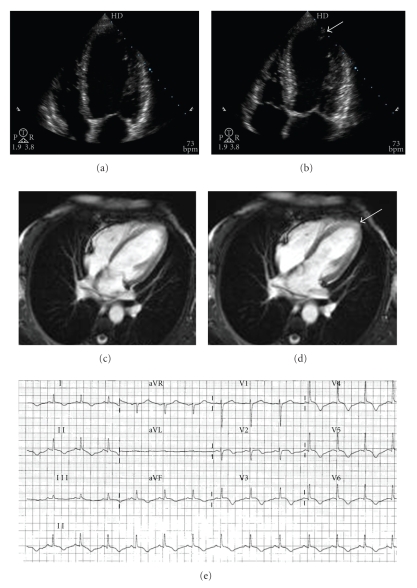
(a) Regional wall-motion abnormality confirmed by transthoracic echocardiography during diastole; (b) systole (arrow); (c) magnetic resonance imaging showing wall motion abnormalities during diastole; (d) systole (arrow); (e) twelve-lead ECG demonstrating resolution of ST-segment elevation, development of T-wave inversion, and prolongation of the QT interval.

Transthoracic echocardiogram showed an abnormal left ventricle with akinesia of apex, anterolateral segments, and interventricular septum (Figures 2(a)  and 2(b)). Left ventricular ejection fraction decreased to 35% whereas right ventricle size and function were normal. No pericardial effusion or pulmonary hypertension was identified. Cardiac magnetic resonance imaging confirmed regional wall-motion abnormalities (Figures 2(c)  and 2(d)) and was helpful in excluding delayed hyperenhancement suggestive of myocardial infarction or myocarditis.

Considering the normality of the patient coronary arteries, we attempted to determine the exact etiology of the present acute cardiomyopathy. We therefore tested blood antibodies directed to cytomegalovirus, Epstein-Barr virus, human immunodeficiency virus, herpes simplex virus 1/2, hepatitis B, C, and A virus, respiratory syncytial virus, enterovirus, adenovirus, and parvovirus to exclude viral myocarditis. Coxiella, chlamydia, borrelia, rickettsia, and Syphilis antibodies were also measured, all of them yielded negative results. Moreover, diagnosis of autoimmune myocarditis was excluded since ACAN, ANCA, and complement C3 and C4 levels were normal. In addition, diagnosis of pheochromocytoma was subsequently excluded by normal levels of metanephrine (plasma levels 23 pg/mL [normal <90 pg/mL], urinary excretion 312 *μ*g/24 hours) and normetanephrine (plasma levels 68 pg/mL [normal <200 pg/mL], urinary excretion 472 *μ*g/l). 

Finally, her clinical status rapidly improved with aspirin, ACE-inhibitor and beta-blockers therapies. Four days later the patient was totally asymptomatic. The ECG at discharge showed prolongation of the **Q** **T** interval associated with a persistent ST-segment elevation and subepicardial ischemia (Figure 2(e)). One month later the patient was under treatment and reported no symptoms with normal hemodynamic status. She exhibited no signs of heart failure and a new echocardiogram showed a total recovery of left ventricular function. She was not recommended to return to playing squash again in a competitive sport league and her treatment was stopped after 3 months.

## 3. Discussion

Diagnosis of Takotsubo cardiomyopathy due to acute physical stress was confirmed in presence of normal coronary arteries and transitory left ventricular dysfunction with typical apical ballooning in the context of a clinical presentation simulating an ACS. ECG features of Takotsubo cardiomyopathy are usually nonspecific and include dynamic ST elevation and/or T-wave inversion typically throughout the anterior leads. Discrepancy between the considerable left ventricle functional abnormalities and the small troponin release in addition to a rapid recovery of her status reinforced the diagnosis.

During squash the body is subject to sudden and vigorous demands inducing a prolonged and severe workload on the myocardium. During exercise, squash player heart rate response may rise from 70 beats/min to more than 150 beats/min in a very short time [1]. As it has been shown by Brady et al., squash in veteran players elicits a dramatic increase in circulating levels of catecholamine, lactate and free fatty acids and that lasts until the early postexercise period [2]. There are cases in literature indicating a pathogenic role of catecholamines in transient left ventricular ballooning syndrome [3]. In our case, adrenergic hypersecretion was suspected, however, we were not able to confirm adrenergic hypersecretion because our measurements were not performed at admission, but later during the hospital stay. Other potential mechanisms of Takostubo have been described such as microvascular abnormalities or epicardial coronary artery spasm, and Takotsubo cardiomyopathy may also reflect stunned myocardium from a neurogenic source [3]. There was no evidence that one of these mechanisms might have been involved in our case. A recent report has also shown transient left ventricular apical ballooning induced by treadmill exercise testing [4]. To our knowledge, this report is the first to describe Takotsubo cardiomyopathy secondary to squash playing occurring in a competitive sport league and in any sport. Finally, regarding long-term management considerations, chronic treatment with beta-blockers, ACE-inhibitors, and aspirin did not provide any benefit in patients with Takotsubo cardiomyopathy [5].

In conclusion, squash and equivalent recreational sports with a risk of adrenergic hypersecretion should be added to the list of causes capable of provoking the Takotsubo syndrome. The underlying pathophysiological mechanisms are unclear at present.
